# Shifts in Chronic Disease Patterns Among Spanish Older Adults With Multimorbidity Between 2006 and 2017

**DOI:** 10.3389/ijph.2023.1606259

**Published:** 2023-10-18

**Authors:** Jeroen J. A. Spijker, Elisenda Rentería

**Affiliations:** Centre d’Estudis Demogràfics, Bellaterra, Spain

**Keywords:** multimorbidities, morbidity, chronic disease, ageing, health inequalities, cluster analysis, principal component analysis, Spain

## Abstract

**Objectives:** To investigate changes in multimorbidity patterns among Spanish older adults.

**Methods:** Data come from the Spanish National Health Survey (ENSE) for individuals aged 60–89 years (2006: *n* = 9,758; 2017: *n* = 8,535). Prevalence rates and relative risks of 20 chronic conditions are estimated for the multimorbidity (3+ chronic conditions) sample, along with observed-to-expected prevalence of three-way disease combinations. Principal component and cluster analyses identify multimorbidity patterns and track temporal changes.

**Results:** Overall, multimorbidity remained stable [2006: 59.6% (95% CI: 58.7%–60.6%); 2017: 60.3% (CI: 59.3%–61.3%)], except at older ages. Women exhibited higher multimorbidity prevalence, but sex differences declined by five percentage points. Low-high education differences widened by three percentage points. In 2017 most individuals living with multimorbidity experienced hypertension (63.4%), osteoarthrosis (62.4%) and chronic back pain (55.9%). These chronic conditions also dominate the most common triadic combinations. Multimorbid men also saw increases in cholesterol and diabetes.

**Conclusion:** Multimorbidity trends and the most common combination of diseases can help plan healthcare for an ageing population. Sex and socioeconomic differences pose additional public health challenges as women and deprived populations tend to have more health complexities.

## Introduction

Life expectancy in Spain has steadily increased in recent decades and now ranks among the world’s highest [1]. As deaths progressively occur at older ages, important changes in the type of diseases and chronic conditions have ocurred. This development is best described by the epidemiological transition theory [2]. A key concern with increasing longevity is whether additional years are spent in good health or if morbidity is becoming more prevalent. To date, in low-mortality countries, no clear pattern has been established between life expectancy and disease- or disability-free life expectancy (DFLE) [3–11]. Results depend on several factors, including the chosen measure for morbidity when calculating DFLE (e.g., disability, chronic conditions) and which country and time period is analysed. In Spain, despite declines in severe disability among older people during the 1990s [10–12], self-care disability among women underwent a sharp rise [10]. From 1997 to 2010, disease presence increased due to earlier age-specific onset and increased life expectancy [13] and the most recent data suggest a continuation of this trend [14].

It appears that declines in mortality have increased the time spent in disability and with chronic diseases. However, when examining the epidemiological transition, the focus tends to be on single cause-of-death groups or the presence of a chronic condition/disease (e.g., [14, 15] for Spain), rather than multiple causes of death or the simultaneous presence of chronic conditions/diseases.

Furthermore, as health inequalities continue to exist between men and women and social classes [16], it becomes crucial to explore, besides the prevalence of specific diseases across sex and socioeconomic groups (SES), the co-occurrence of chronic conditions. This is because multimorbidity is strongly associated with increased frailty, risk of disability and, therefore, more healthcare utilisation and risk of death [17]. In this context, despite Spain’s high life expectancy, adults aged 70 years and older exhibited higher rates of multimorbidity compared to other European countries in 2011 [18].

Multimorbidity studies often adopt varying approaches. Some exclusively focus on the coexistence of two diseases/chronic conditions (henceforth comorbidity) or three or more diseases/chronic conditions (henceforth multimorbidity) in different regions and from different surveys. These studies analyse age and cohort patterns, socioeconomic inequalities or link multimorbidity to quality of life and healthcare use [19–25]. In contrast, other studies emphasize the nature of associations among diseases, necessitating the application of distinct cluster or factor analysis methods to discern the most prevalent disease combinations [25–29].

Regarding common co- and multimorbidity patterns, Garin et al [28] identified distinct patterns in the population aged 50 years and older across nine countries, focussing on 12 highly prevalent chronic conditions. Hypertension emerged as the most prevalent comorbid condition, especially among individuals with obesity, stroke, diabetes, and angina. Arthritis and cataracts were also common comorbid conditions. In Spain, analysis of data from the COURAGE project revealed the presence of a “cardio-respiratory” (angina, asthma, COPD) and “metabolic” (diabetes, obesity, hypertension) pattern. Another study using the same dataset [27], which also included anxiety disorders in the analysis, highlighted a clear “mental-articular” pattern (arthritis, depression, anxiety), possibly due to the psychological burden of arthritis [30].

Concerning SES differentials in multimorbidity, studies from other countries have identified a gradient similar to that of mortality, with a higher prevalence of more than two diseases among lower socioeconomic groups [31–33]. However, a similar study in Spain is needed to better understand which diseases are more affected by SES.

In this context, our paper’s main objective is to analyse trends in multimorbidity prevalence among older people, defined here as the presence of at least three chronic conditions among 60–89 years-olds living in Spain in 2006 and 2017. We also take special interest in assessing sex, age and socioeconomic health differences and whether there have been changes in the patterns of co-occurring common chronic diseases over time. Similar to Islam et al. [34], we apply different methods to a single dataset as there is no consensus in existing literature on how to measure the co-occurrence of diseases. However, if our results exhibit a high degree of consistency, it would provide confidence in the observed patterns. Specifically, we first identify the most important combinations of three chronic conditions (referred to as triadic combinations) and then employ cluster analysis and principal component analysis on 20 chronic conditions among the older population living with multimorbidity.

## Methods

### Study Design and Population

Data on the Spanish population aged 60–89 years are extracted from the 2006 and 2017 Spanish National Health Surveys (ENSE), with sample sizes of 9,758 and 8,535, respectively. The ENSE is a cross-sectional survey conducted roughly every 5 years on a representative sample of the non-institutionalised population. In 2006, a comparable list of chronic conditions was introduced, allowing for comparisons with the most recent data available from the same survey. Individuals were considered to have a chronic condition if they reported being diagnosed with and suffering from the condition during the last 12 months. We define multimorbidity as the presence of at least three chronic conditions from the list of 20 conditions used in our analysis (see Supplementary Table S1 for sample description and Table 1 for the list of chronic conditions). This threshold has also been used in other studies [34–36].

**TABLE 1:** List of chronic conditions used in the analyses according to prevalence rank order of the total sample in 2017 (%), the prevalence in the total sample, the multimorbidity sample and the relative risk for multimorbidity, 2006 and 2017 by gender for ages 60–89 (Spain. 2006 and 2017).

	Chronic condition	Total				Men				Women				Total		Men		Women	
		2006		2017		2006		2017		2006		2017		2006	2017	2006	2017	2006	2017
		TS	MMS	TS	MMS	TS	MMS	TS	MMS	TS	MMS	TS	MMS	RR	RR	RR	RR	RR	RR
1	Hypertension	45.0	57.9	47.7	63.4	39.7	54.4	47.6	66.3	49.3	60.0	47.8	61.5	2.2	2.7	2.2	2.5	2.3	3.0
2	Osteoarthrosis	49.2	69.5	43.1	62.4	33.7	54.8	27.9	44.6	61.6	78.0	55.9	74.4	3.6	4.6	4.2	5.0	2.9	3.9
3	Cholesterol	27.5	38.5	38.5	52.4	24.3	37.3	38.0	55.2	30.0	39.3	38.8	50.4	3.5	3.0	3.2	3.0	3.7	3.2
4	Chronic back pain	38.5	57.8	37.5	55.9	27.4	46.5	28.1	44.7	47.5	64.3	45.5	63.3	5.8	5.8	5.4	4.8	5.5	6.3
5	Obesity	23.0	31.0	23.8	32.8	21.0	31.1	22.5	32.5	24.6	30.9	24.9	32.9	2.7	3.1	2.9	3.0	2.6	3.3
6	Diabetes	16.1	21.8	20.5	29.5	17.3	26.1	22.9	35.2	15.1	19.3	18.4	25.6	2.8	4.3	3.0	3.9	3.0	6.0
7	Varicose veins	20.8	30.9	17.4	26.2	10.3	17.6	8.7	13.9	29.2	38.6	24.7	34.5	5.3	6.8	5.6	4.8	4.2	6.8
8	Mental health problems	17.6	26.8	16.1	24.3	9.4	16.6	8.8	14.3	24.2	32.8	22.2	31.0	6.9	6.7	7.0	5.5	5.7	6.4
9	Heart disease	13.7	20.3	14.9	21.6	14.6	24.3	16.5	26.0	12.9	18.0	13.5	18.6	5.2	4.6	4.7	4.6	8.3	5.4
10	Allergies	8.1	12.2	12.5	18.2	6.1	10.3	9.2	14.6	9.7	13.3	15.3	20.7	6.0	4.8	5.2	4.7	6.3	4.5
11	Urinary incontinence	10.3	16.0	11.5	18.2	9.3	16.9	9.8	17.0	11.0	15.5	13.0	18.9	9.2	**12.6**	9.1	**10.2**	9.8	**16.2**
12	COPD/asthma	11.7	17.3	10.4	15.4	13.9	23.1	11.0	17.5	10.0	13.9	9.8	14.0	4.9	5.6	4.7	4.8	7.6	8.4
13	Osteoporosis	13.7	21.7	10.2	15.6	3.6	7.0	2.1	3.8	21.7	30.3	17.0	23.5	**13.1**	7.9	**29.1**	**16.5**	8.7	5.8
14	Prostate	9.3	13.1	8.6	12.2	21.0	35.6	18.8	30.3	—	—	—	—	3.5	3.8	5.3	5.1	—	—
15	Thyroid dysfunction	4.6	6.8	8.1	11.8	1.1	1.9	2.5	4.0	7.5	9.7	12.9	17.0	5.1	4.7	6.3	5.6	3.7	3.6
16	Haemorrhoids	9.3	14.5	7.9	12.4	7.8	14.2	6.0	10.4	10.5	14.7	9.6	13.7	8.8	**10.4**	9.0	9.3	8.6	**10.9**
17	Migraine	10.7	16.8	7.5	11.3	6.1	11.0	4.1	6.8	14.3	20.2	10.3	14.3	**11.0**	6.6	7.9	6.0	11.9	6.0
18	Chronic skin problems	7.0	10.9	7.4	11.4	7.8	14.3	7.4	12.6	6.4	8.9	7.5	10.6	8.4	8.1	**10.4**	8.3	7.5	8.3
19	Constipation	10.2	15.9	7.2	11.4	6.6	11.0	3.7	6.6	13.1	18.7	10.1	14.6	8.4	**14.1**	4.8	**15.8**	**13.7**	**11.5**
20	Gastric and duodenal ulcer	6.0	8.8	4.2	6.5	6.0	9.5	3.9	6.4	6.1	8.4	4.5	6.5	4.5	8.2	3.9	5.9	6.6	**14.7**

Notes: TS, total sample; MMS, multimorbid sample; RR, relative risk. RRs equal or greater than 10.0 are printed in bold. These chronic conditions have been diagnosed by a medical doctor and the respondent experienced during the last 12 months. The exception is obesity, as this was calculated on the basis of weight and height of the respondent, taking a BMI of at least 30 as the definition of obesity. Respondents are 60–89 years old. Regarding mental health problems [8], in 2006 these pertain to the chronic condition category “depression, anxiety or other mental illnesses.” In 2017 this was split up into separate categories (“depression,” “anxiety,” “other mental illnesses”). Hence, in order to establish a comparable list of health conditions for the analysis, the three distinct mental health categories from the 2017 data were combined into one, thus treating respondents who reported multiple mental health issues as having only one mental health problem. However, considering the congruence of these categories with the 2006 classification and the marginal shifts in proportions over time (see table), results are unlikely to be affected. Lastly, ENSE also asked respondents about several other chronic conditions, including stroke, cancer and menopause, but as they had a prevalence too we considered too low for our triadic combination analysis (<4%) we did not analyse them. Neither did we analyse cataracts despite a 16% prevalence in 2017 because of the likelihood of being cured if operated (meaning that it is no longer a chronic condition). Data source: the Spanish National Health Survey (ENSE).

### Outcome Variables

For each chronic condition, prevalence rates were calculated for the multimorbidity sample (mm-sample) as well as the Relative Risk (RR). RR represents the likelihood of multimorbid persons managing a specific chronic condition compared to persons without multimorbidity but who live with the same condition [35]. To investigate the independence of diseases, observed-to-expected prevalence ratios (O/E) were calculated for all triadic combinations. The observed prevalence was obtained from the survey, and the expected prevalence was estimated by multiplying the prevalence of each of the three single conditions. An O/E ratio greater than 1 indicates that diseases are not independent, as the combination occurs more frequently than expected. Sample weights were applied to all prevalence estimates to account for age, sex and regional population structure. The prevalence of multimorbidity and triadic disease combinations was computed for men, women, 10 years age groups (60–69, 70–79, 80–89) and educational attainment (less than primary school, primary and lower secondary school, upper secondary, vocational training and university).

### Statistical Analysis

To facilitate our aim of identifying meaningful groups of co-occurring chronic conditions, we exclusively analysed the multimorbidity sample (mm-sample). To enhance the reliability of our findings, we employed two methods:

i) Principal component analysis (PCA) of the 20 chronic conditions with a varimax rotation to simplify the complex relationships among the different chronic conditions (variables) into a reduced set of factors (principal components). Each component is a weighted sum of the original variables, where the weights (factor loadings) indicate the correlations between the variable and the principal component. Factors were selected based on eigenvalues greater than one and variable factor loadings greater than 0.3. Components were ordered by their ability to explain the maximum possible variance. The component with the highest eigenvalue was considered the most important in explaining the observed data variation [37].
ii) Agglomerative hierarchical cluster analysis, based on the relative proximity of the 20 chronic conditions. The conventional approach to cluster analysis classifies *n* respondents into clusters based on proximity, producing an *n* × *n* proximity matrix reflecting the degree of closeness among respondents. However, it is also possible to produce multimorbidity groups (clusters) based on the presence or co-occurrence of two or more chronic conditions in a single individual by reducing the data matrix to a much smaller *p* × *p* proximity matrix of chronic conditions [34, 38]. Following the methodology applied by Islam et al [34], we used Yule’s Q as the similarity measure. To measure distance between two clusters, we opted for centroid linkage as it yielded a clustering solution similar to the components obtained from PCA. Analyses were performed using SPSS version 24.

## Results

### Time Changes in Prevalence Rates in Multimorbidity According to Sociodemographics

**FIGURE 1:**
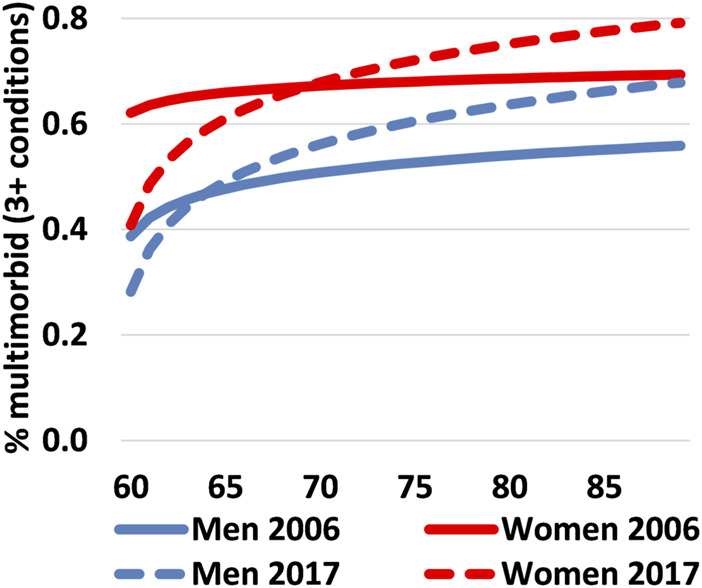
Proportion of the Spanish population aged 60–89 years who live with multimorbidity by single age & sex, Spain 2006 vs. 2017. Note: The age-specific proportions were fitted in EXCEL using a logarithmic equation. Data source: the Spanish National Health Survey (ENSE).

**FIGURE 2:**
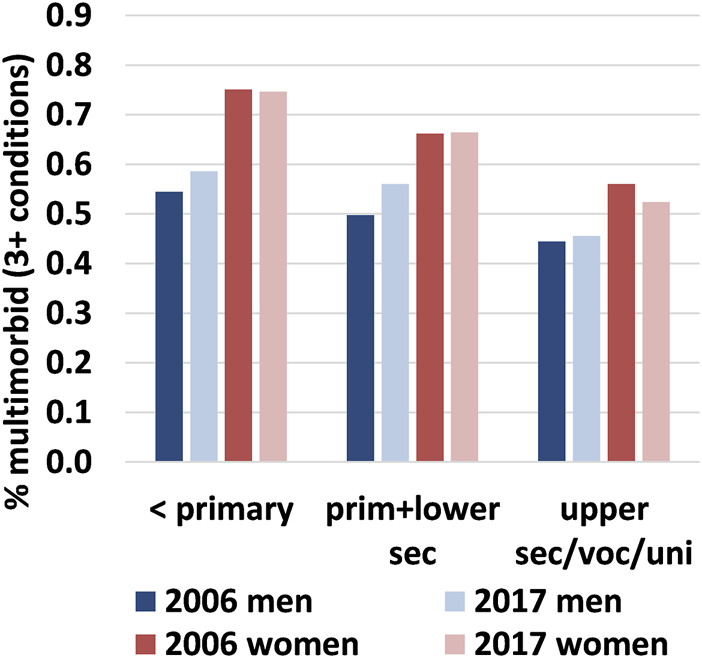
Proportion of the Spanish population aged 60–89 who live with multimorbidity by sex & educational attainment, Spain 2006 vs. 2017. Note: The proportions are age-standardised to take into account the educational expansion that took place in Spain between the late 1950s and 1970s (i.e., correcting for the fact there are more older people who had not completed primary school in 2006 than in 2017, who are also more likely to live with 3+ conditions). This permits results to be compared over time. Data source: the Spanish National Health Survey (ENSE).

Between 2006 and 2017, there was no overall change in the prevalence of multimorbidity for people aged 60–89 years. In both years, 6 out of every 10 people had at least three chronic conditions [2006: 59.6% (95% CI: 58.7%–60.6%); 2017: 60.3% (CI: 59.3%–61.3%)]. The prevalence of multimorbidity was notably lower for men, although the sex gap reduced by five percentage points over time. Prevalence among men increased by three percentage points to 53.1% (95% CI: 51.6%–54.7%), while among women it decreased by two percentage points to 66.3% (95% CI: 65.0%–67.7%). Regarding age, multimorbidity was higher in 2017 than in 2006 among men aged 64 years and older and women aged 70 years and older. However, below these ages, multimorbidity declined (Figure 1). Changes were not uniform across educational levels and sex as increases over time occurred mainly among lower-educated men, while among higher-educated Spanish women there was a slight decline between periods (Figure 2).

We then estimated the average number of conditions among individuals with and without multimorbidity by sex and educational level and compared their trend over time (Table 2). Results showed that among those living with at least three chronic conditions, women managed on average 0.7 chronic conditions more than men did in 2006 [women 5.34 (95% CI: 5.27–5.40); men: 4.64 (95% CI: 4.60–4.67)] and 0.8 more in 2017 [women 5.41 (95% CI: 5.33–5.48); men: 4.60 (95% CI: 4.57–4.64)]. For both sexes the higher the educational level, the fewer chronic conditions. There were no statistically significant increases in the number of chronic conditions among multimorbid individuals between the two periods, irrespective of their educational category. For individuals without multimorbidity, there were no statistically significant differences in the average number of conditions in the two survey years, regardless of sex and educational attainment. The only exceptions were in 2006 [all women: 1.30 (95% CI: 1.22–1.38); all men 1.15 (95% CI: 1.11–1.18)] and for those with less than primary school [1.12 (95% CI: 1.04–1.19) for men and 1.38 (95% CI: 1.21–1.54) for women]. Age differences were also analysed, but no or only minor increases over age were discerned. For instance, among men with less than primary school, 60–64 years-olds had an average of 1.25 chronic conditions compared to 1.19 for 85–89 years-olds. The corresponding figures for low-educated women were 1.29 and 1.44, respectively. For those with upper secondary school and higher education, the figures were 1.04 and 1.24 for 60–64 and 85–89 years-old men and 0.93 and 1.37 chronic conditions for 60–64 and 85–89 years-old women (see Supplementary Figure S1).

**TABLE 2:** Average number of conditions together with 95% confidence intervals among persons who live without/with multimorbidity by sex and educational level (age-standardized) (Spain. 2006 and 2017).

	Men				Women			
	Multimorbidity				Multimorbidity			
	No		Yes		No		Yes	
	2006	2017	2006	2017	2006	2017	2006	2017
**Total**	**1.15**	**1.17**	**4.64**	**4.60**	**1.30**	**1.19**	**5.34**	**5.41**
	1.11–1.18	1.14–1.21	4.60–4.67	4.57–4.64	1.22–1.38	1.12–1.27	5.27–5.40	5.33–5.48
Less than primary school	1.12	1.21	4.94	5.05	1.38	1.32	5.67	5.83
	1.04–1.19	1.12–1.31	4.89–5.00	4.97–5.13	1.21–1.54	1.14–1.49	5.57–5.77	5.69–5.97
Primary/lower sec school	1.20	1.20	4.59	4.52	1.29	1.19	5.21	5.26
	1.15–1.25	1.15–1.25	4.55–4.64	4.47–4.57	1.19–1.40	1.08–1.30	5.12–5.29	5.16–5.36
Upper sec/vocational/university	1.11	1.15	4.36	4.46	1.20	1.10	4.76	5.03
	1.03–1.18	1.08–1.21	4.27–4.45	4.38–4.53	1.02–1.39	0.96–1.25	4.56–4.96	4.86–5.20

“Total” written in bold. Data source: the Spanish National Health Survey (ENSE).

### Prevalence of Chronic Diseases and the Relative Risks of Multimorbidity

Table 1 presents the prevalence of the chronic conditions for both the total and multimorbidity samples. In 2017, the highest prevalences among the multimorbidity sample in 2017 were observed for hypertension (63.4%), osteoarthrosis (62.4%), chronic back pain (55.9%) and cholesterol (52.4%). Cholesterol exhibited the largest increase since 2006 (+13.9 percentage points/36% higher). Regarding sex differences and changes over time, men showed the largest increases in cholesterol (+17.9 percentage points/48% higher), followed by hypertension (+11.9 percentage points/22% higher) and diabetes (+9.1 percentage points/35% higher). Among women, remarkable increases were noted for cholesterol (+11.1 percentage points/28% higher), allergies (+7.4 percentage points/56% higher) and thyroid dysfunction (+7.3 percentage points/75% higher). Concomitantly, noteworthy declines were observed in osteoarthrosis among men (−7.1 percentage points/19% lower) and osteoporosis among women (−6.8 percentage points/22.4% lower). The table also shows the RR for specific chronic conditions by comparing the prevalence of each condition between the mm- and non-mm samples. Despite similarity in ranking in both years, noteworthy is the increase in RR for multimorbidity between 2006 and 2017 for constipation, especially for men (from 4.8 to 15.8), while marked increases among women were observed for urinary incontinence (from 9.8 to 16.2) and duodenal ulcers (from 6.6 to 14.7).

### Prevalence of Triadic Combinations

Our next step involved analysing the triadic combinations of chronic conditions among multimorbid individuals (Table 3). The 20 selected conditions provided us with 20!/(17!·3!) = 1,140 combinations. After sorting them according to frequency of occurrence in 2017 and considering only the 10 top-ranked combinations for the total population aged 60–89 years, we observe that triadic combinations involving osteoarthrosis [8], hypertension [6] and back pain [6] predominated in 2017. This pattern remained stable between 2006 and 2017 among women, however, among men, there was a clear shift towards more multimorbidity associated with hypertension (+4), cholesterol (+4) and diabetes (+2). Further analysis considering education levels revealed that a higher proportion of older men living with multimorbidity experienced problems associated with the prostate in 2006 compared to 2017. Regarding women, bone diseases, back pain, varicose veins and mental health issues remained important conditions in the multimorbidity patterns over time, especially for those with lower secondary school or less (Supplementary Table S2).

**TABLE 3:** Prevalence (prevalence rank) and observed/expected ratio of the most common 3-way disease combinations of chronic conditions among 60–89 years-old people living with multimorbidity in Spain, 2006 and 2017. Total and by sex.

Combination	Total		Men		Women		Total		Men		Women	
	2006	2017	2006	2017	2006	2017	2006	2017	2006	2017	2006	2017
	Prevalence (rank)		Prevalence (rank)		Prevalence (rank)		O/E (Rank)		O/E (Rank)		O/E (Rank)	
Hypertension & osteoarthrosis & back pain	**25.1 (1)**	**25.2 (1)**	**15.2 (1)**	**15.4 (3)**	**30.9 (1)**	**31.8 (1)**	1.1	1.1	1.1	1.2	1.0	1.1
Hypertension & cholesterol & osteoarthrosis	**16.0 (3)**	**21.0 (2)**	11.0 (4)	**15.5 (2)**	18.9 (6)	**24.7 (3)**	1.0	1.0	1.0	0.9	1.0	1.1
Cholesterol & osteoarthrosis & back pain	**17.6 (2)**	**20.3 (3)**	**11.7 (2)**	13.0 (6)	**21.1 (3)**	**25.2 (2)**	1.1	1.1	1.2	1.2	1.1	1.1
Hypertension & cholesterol & back pain	12.2 (10)	18.1 (4)	8.1 (8)	15.2 (4)	14.7 (12)	20.1 (4)	0.9	1.0	0.8	0.9	1.0	1.0
Hypertension & cholesterol & diabetes	6.6 (43)	14.3 (5)	6.2 (21)	**17.9 (1)**	6.8 (64)	11.8 (19)	1.3	**1.5**	1.2	1.4	**1.5**	**1.5**
Osteoarthrosis & back pain & varicose veins	15.9 (4)	13.9 (6)	5.8 (26)	5.3 (38)	**21.8 (2)**	19.8 (5)	1.3	**1.5**	1.3	**1.9**	1.1	1.2
Obesity & osteoarthrosis & back pain	13.8 (7)	13.2 (7)	8.9 (7)	7.5 (18)	16.7 (8)	17.0 (7)	1.1	1.2	1.1	1.2	1.1	1.1
Osteoarthrosis & back pain & mental health	15.2 (5)	13.0 (8)	6.3 (20)	4.4 (55)	20.3 (5)	18.9 (6)	1.4	**1.5**	**1.5**	**1.6**	1.2	1.3
Hypertension & Obesity & osteoarthrosis	13.7 (8)	13.0 (9)	9.9 (5)	8.8 (11)	15.9 (9)	15.9 (10)	1.1	1.0	1.1	0.9	1.1	1.1
Hypertension & diabetes & osteoarthrosis	9.3 (17)	12.2 (10)	6.7 (15)	9.8 (9)	10.7 (22)	13.8 (12)	1.1	1.0	0.9	0.9	1.2	1.2
Osteoporosis & back pain & prostate (men)	—	—	**11.7 (3)**	7.3 (20)	—	—			1.3	1.2		

Notes: O/E, observed/expected prevalence (see main text). The three most prevalent triads/highest O/E ranks in each year are printed in bold. Data source: the Spanish National Health Survey (ENSE).

Table 3 also displays the estimated O/E ratio for the most frequent triadic combinations of chronic conditions. Notably, suffering from the three most frequent triadic combinations (hypertension/osteoarthrosis/back pain, hypertension/cholesterol/osteoarthrosis, and cholesterol/osteoarthrosis/back pain) is approximately as expected based on the prevalence of the individual conditions (O/E ratio between 0.9 and 1.2). On the other hand, the O/E ratio of men suffering from osteoarthrosis and backpain along with either mental health or varicose veins equaled, respectively 1.6 and 1.9 in 2017, suggesting that these chronic diseases are not independent of each other. Both O/E ratios also increased over the studied period. For women, the observed risk of suffering simultaneously from hypertension, cholesterol and diabetes is 1.5 higher than the expected risk. Worth mentioning is that the majority of triads with a high O/E ratio consists of combinations of chronic diseases with relatively low prevalence. In the 2017 data, none of the ten triadic combinations with the highest O/E ratios belong to the 100 most prevalent combinations among individuals living with multimorbidity. Conversely, all ten triadic combinations with highest O/E ratios include constipation as one of the chronic conditions, six include hemorrhoids and four involve gastric ulcers. The combination of haemorrhoids/migraine/constipation holds the highest O/E ratio of 5.8 (Supplementary Table S3).

### Principal Component and Cluster Analyses

**FIGURE 3:**
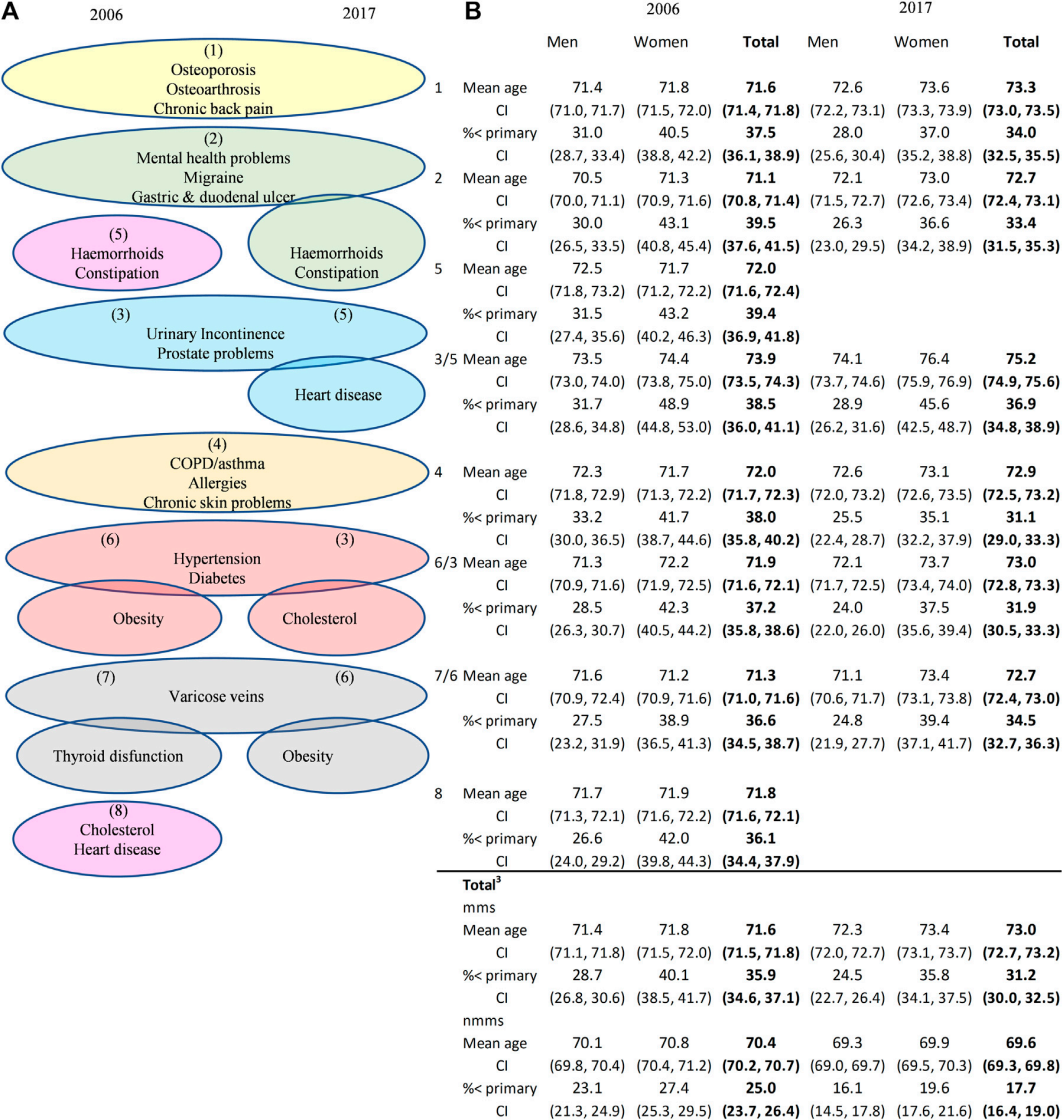
PCA-derived components^1^ and chronic diseases with the highest factor loadings **(A)** and sex-specific, mean age and the proportion with less than primary school education of the total multimorbidity sample, the non-multimorbidity sample and those in the mms who are living with at least one chronic disease listed under each component **(B)**, Spain 2006 vs. 2017^3^. Note: 1. The order of the components are shown in brackets. No chronic disease had a factor loading of ≥0.3 in more than one component. 2. All loadings for thyroid dysfunction in 2017 were <0.3. 3. Total summary statistics for the multimorbid (mms) and non-multimorbid (nmms) samples pertain to the weighted population aged 60–89. Data source: the Spanish National Health Survey (ENSE).

Lastly, on the group of participants with multimorbidity (mm-sample) we performed PCA and cluster analyses on the 20 single conditions to identify frequently co-occurring conditions. For 2006, the PCA derived eight components with eigenvalues exceeding 1, explaining almost half of the variation in chronic conditions (Supplementary Figure S2 and Supplementary Table S4). The first component exhibited particularly high factor loadings for osteoarthrosis and chronic back pain and, to a lesser extent, osteoporosis. Most other components also contained closely-related chronic conditions with high factor loadings. For instance, component 3 contained urinary incontinence and prostate problems, component 4 COPD/asthma and allergies (and to a lesser extent chronic skin problems) and component 5 haemorrhoids and constipation. Regarding 2017, only six components had eigenvalues exceeding 1, which collectively explained 40.4% of the variance. However, these components exhibited substantial overlap with the situation in 2006 regarding the most important chronic conditions they represented. As shown in Figure 3, the first and fourth components for both years contain the same three chronic conditions that were selected for each component based on their high factor loadings (>0.3), as indicated in Supplementary Table S4. Component 2 in 2017 has the same bowel-related issues and mental health conditions as in 2006, but with the addition of haemorrhoids and constipation. The third component includes hypertension, cholesterol and diabetes, conditions that appeared less important among multimorbid individuals in 2006.

To determine sex, age and educational differences in the disease patterns of the population living with multimorbidity, the table in Figure 3 shows the characteristics of those who manage one or more of the chronic diseases listed under each component. For instance, individuals suffering from osteoporosis, osteoarthrosis and/or chronic back pain (listed under component 1) have similar demographic characteristics as the general population living with multimorbidity aged 60–89 years. Conversely, those with mental health issues are, on average, up to 1 year younger (statistically significant at the 5% level for men in 2006), while people living with multimorbidity and managing urinary incontinence and prostate problems (component 3 in 2006) are 2–3 years older. Regarding changes over time, the mean age of the male sample living with multimorbidity increased by 0.9 years, and for women with multimorbidity by 1.7 years. Concurrently, the male and female populations with fewer than three chronic conditions were, on average, 0.8 and 0.9 years younger in 2017 compared to 2006. This suggests that not only is multimorbidity not being delayed but that over the course of the decade, increases in life expectancy were accompanied by increases in the time lived with multimorbidity.

As for the cluster analyses we conducted (Supplementary Table S5 and Supplementary Figure S3), the results closely mirrored the components derived from the PCA. In the dendrogram for the 2006 data, the first cluster included haemorrhoids, constipation, gastric duodenal ulcer, mental health problems and migraine‒the main chronic conditions represented in component 2 and 5. Likewise, the next cluster (thyroid dysfunction and varicose veins) corresponded to component 7 and cluster 3 (osteoarthrosis, osteoarthritis and chronic back) to component 1 of the PCA. Cluster 4 and PCA component 4 were identical (COPD, allergies and chronic skin problems), while cluster 5 coincided with component 6 in the case of hypertension, diabetes and obesity but also included cholesterol and heart disease. Obesity was the last chronic condition added to cluster 5, but given the commonality (all are cardiovascular disease risk factors), the cluster made sense from an epidemiological perspective. The same applied to the last cluster (urinary incontinence and prostate problems). Turning to the 2017 results, not all clusters remained identical: obesity did not join hypertension, diabetes and cholesterol, while the cluster with urinary incontinence and prostate problems included heart disease (which make clinical sense for men as these three chronic diseases had an O/E of 1.9; results available upon request). Lastly, the first cluster included both chronic intestinal problems (haemorroids, constipation, gastric and duodenal ulcer, varicose veins), metabolic issues (thyroid dysfunction) as well as mental health problems and migraine, almost coinciding with PCA components 3 and 7).

## Discussion

In this study, we analysed the prevalence of multimorbidity (defined as suffering from at least three chronic conditions) among the 60–89 years-old population in Spain in 2006 and 2017, focusing on 20 chronic conditions. Overall, we found that multimorbidity did not increase, as it affected six out of ten older people in both years. However, there were some notable trends: prevalence increased among men after age 64 and among women after age 70. Importantly, the average number of conditions among older adults with multimorbidity did not increase. Regarding sex differences, in both 2006 and 2017, women with multimorbidity encountered, on average, over half a chronic condition more than men. However, while the prevalence of multimorbidity among men increased by three percentage points, it decreased among women by two percentage points. Educational differences followed the expected pattern, with higher-educated individuals having fewer conditions than the lower educated. Nevertheless, educational differences in the presence of multimorbidity widened over time. The prevalence of multimorbidity was highest for those suffering from hypertension, osteoarthrosis and chronic back pain (63.4%, 62.4%, and 55.9%, respectively, in 2017). However, the relative risk for multimorbidity was highest for digestive-related conditions, especially constipation and urinary incontinence (14.1 and 12.6, respectively, in 2017).

In 2017, the most common triadic combinations of chronic conditions among multimorbid individuals included osteoarthrosis, hypertension and/or back pain. However, when it comes to men, they also experienced increases in cholesterol and diabetes, alongside a slight decline in chronic prostate problems between 2006 and 2017. For instance, the triad of osteoporosis/back pain/prostate was ranked third in 2006 but 20th in 2017. Concurrently, the number of triadic combinations of chronic conditions that included prostate problems within the top 100 dropped from 30 to 21 during the same period. Possible reasons include both better treatment and fewer diagnoses of prostate disorders due to new recommendations in prostate cancer screening in older men [39, 40]. Among multimorbid women, triadic combinations including osteoarthrosis, back pain and/or hypertension were most prevalent, with little change over time. It is also worth noting that the most frequent triadic combinations closely align with what we would anticipate from the prevalence of individual conditions (i.e., the O/E ratios are approximately 1). Conversely, combinations that show interdependence (i.e., high O/E ratios) exhibit relatively low prevalence, a trend resembling the findings in Germany [35] concerning triadic combinations among health insurance policy holders aged 65 years and older.

Comparing our findings with other studies directly is challenging due to a multitude of factors impacting the results, including the number and types of diseases considered, sample demographics, underlying risk factors, and data collection methods. Nonetheless, by employing various methodological approaches [34] in which we have demonstrated the importance of similar combinations of chronic conditions in different ways, our study still offers valuable insights for policymakers. Notably, two multimorbidity patterns identified in 2006 remained unchanged in 2017 according to both the PCA and cluster analyses: 1) musculoskeletal disorders (osteoarthrosis, osteoarthritis and chronic back pain), which tend to increase with age [41]; and 2) environment-related chronic diseases (COPD, allergies and chronic skin problems), known to be linked to increased air pollution resulting from rapid urbanization and industrialization [42]. Regarding the remaining chronic conditions, two other multimorbidity patterns can be identified based on similar cluster and PCA outcomes: 3) cardiovascular disease and associated risk factors: Hypertension, diabetes, obesity, cholesterol and heart disease were one cluster and confined to two PCA components in 2006. In 2017 they were more dispersed, with the cardiometabolic risk factors hypertension, diabetes and cholesterol all in one cluster and one PCA component. The same applies to obesity, while heart disease formed a cluster and PCA component with prostate problems and urinary incontinence (known correlates of cardiovascular disease in elderly men [43]). 4) Brain-gut comorbidity (one cluster in both years, but two PCA components in 2006 and one in 2017), containing gastrointestinal problems (haemorrhoids, constipation, gastric duodenal ulcer), together with mental health problems and migraine. These findings align with previous research indicating a connection between gastrointestinal disorders and psychological symptoms [44]. Also noteworthy is that all ten triadic combinations with the highest O/E ratios include constipation, six include hemorrhoids and four involve gastric ulcers. This suggests that individuals with health issues related to the digestive system are susceptible to experiencing various related chronic conditions simultaneously.

Our study has also revealed noteworthy and novel findings when comparing different periods and analysing sex and educational differences. The observed sex gaps reinforce previous literature on the poorer health experienced by women. However, there are indications of a slight improvement among women, marked by a 2-percentage-point reduction in the prevalence of multimorbidity, while men experienced a 3-percentage-point increase. This warrants closer examination in future research. Recognising educational inequalities in multimorbidity is important, as socioeconomic differences often result in deprived populations, particularly those with lower educational attainment, experiencing greater health complexities. This underscores the significance of addressing and reducing social inequalities in health and identifying groups that require special attention from public health services. For instance, a recent study conducted in the United Kingdom, which analysed various determinants of multimorbidity, indicated that the elevated risk of multimorbidity among lower-educated individuals could be partially attributed to their higher BMI and increased smoking rates [45].

This study has certain limitations related to the data and methodology employed in the analysis. Firstly, because it is a cross-sectional analysis, individuals cannot be followed over time, thus precluding discussions about individual changes in multimorbidity prevalence. Secondly, it is important to acknowledge that changes in disease prevalence may also result from improvements in diagnostic practices and alterations in medical protocols. Notably, revisions in recommendations for low-density lipoprotein (LDL) levels in 2011 [46] and 2016 [47] contributed to increased diagnosis of high cholesterol. Additionally, the Spanish Health Surveys only include individuals living in private households, excluding institutionalised individuals with likely poorer health statuses. While this exclusion could impact multimorbidity prevalence rates at older ages, it is worth noting that Spain’s institutional arrangements encompass a relatively low proportion of the population. In 2011, only 7% of individuals aged 85–89 years were institutionalised, with even lower rates in younger age brackets [48].

Studying changes in the prevalence and patterns of chronic diseases linked to multimorbidity is important from a healthcare perspective. This is particularly relevant as health systems remain largely single-illness oriented, despite the complex needs of patients with multimorbidity that are often not addressed [34]. Furthermore, the persistence of disease clusters within multimorbidity patterns observed over time underscores the significance of addressing diseases through a holistic approach rather than treating each chronic condition in isolation. Such an approach can prove highly effective in health prevention, enabling us to anticipate and mitigate future related issues. Considering the increasing number of people experiencing health problems, driven in part by the babyboom generation entering old age, the pressure on the healthcare system is expected to intensify in the coming decades. Therefore, gaining a comprehensive understanding of change in disease prevalence, multimorbidity and the sex and socioeconomic changes therein is crucial for future healthcare planning in an ageing population.
