# Supplementary material for: Shifts in Chronic Disease Patterns Among Spanish Older Adults With Multimorbidity Between 2006 and 2017
Source: Int J Public Health. 2023 Oct 18;68:1606259. doi: 10.3389/ijph.2023.1606259 (PMC10618995; doi:10.3389/ijph.2023.1606259)
Supplement: Supplementary file 1 [file DataSheet1.PDF]

Supplementary material for the article

Spijker J.J.A. and Renteria E. (2023). Shifts in chronic disease patterns among Spanish older adults with multimorbidity between 2006 and 2017. *International Journal of Public Health.*

Supplementary Table S1. Weighted characteristics of the 2006 and 2017 Spanish Health Surveys used in the analysis. Non-institutionalised population aged 60-89.

Supplementary Table S2. Most prevalent 3-way disease combination (% and rank) among 60-89 year-old people living with multimorbidity in 2017, compared with 2006, by sex, sex and age, and sex and education.

Supplementary Table S3. Ten-highest Observed/Expected (O/E) ratios of 3-way disease combinations of chronic conditions and their prevalence and rank among 60-89 year-old people living with multimorbidity in Spain. 2006 and 2017.

Supplementary Table S4. Agglomeration schedule of the hierarchical clustering using centroid linkage and Yule's Q similarity

Supplementary Figure S1. Average number of conditions among persons living without and with multimorbidity by sex, age and educational level. 2006 and 2017.

Supplementary Figure S2. Scree plots of PCA analysis. 2006 and 2007.

Supplementary Figure S3. Dendrogram using centroid linkage and Yule's Q similarity

Supplementary Table S1 Weighted characteristics of the 2006 and 2017 Spanish Health Surveys used in the analysis. Non-institutionalised population aged 60-89.

	Total		Men		Women	
	2006	2017	2006	2017	2006	2017
Sex						
Men	44.5%	45.8%				
Age						
60-69 yrs	45.0%	45.5%	46.6%	48.5%	43.7%	43.0%
70-79 yrs	38.6%	33.6%	38.0%	33.9%	39.2%	33.4%
80-89 yrs	16.4%	20.9%	15.4%	17.6%	17.2%	23.6%
Educational attainment						
less than primary school	31.5%	25.9%	25.9%	20.6%	36.0%	30.3%
primary school+lower sec	52.0%	49.4%	51.9%	48.9%	52.1%	49.7%
upper sec/vocational/higher	16.5%	24.8%	22.3%	30.5%	11.9%	20.0%
3+ chronic conditions	59.6%	60.3%	49.4%	53.1%	67.8%	66.3%
**N***	**9758**	**8535**	**3560**	**3667**	**6198**	**4868**

Note: *Before applying sample weights.

Supplementary Table S2. Most prevalent 3-way disease combination (% and rank) among 60-89 year-old people living with multimorbidity in 2017, compared with 2006, by sex, sex and age, and sex and education.

Combination	Total		Men		Women		Men						Women					
							60-69		70-79		80-89		60-69		70-79		80-89	
	2006	2017	2006	2017	2006	2017	2006	2017	2006	2017	2006	2017	2006	2017	2006	2017	2006	2017
hypertension&osteoarthrosis&back pain	**25.1 (1)**	**25.2 (1)**	**15.2 (1)**	**15.4 (3)**	**30.9 (1)**	**31.8 (1)**	**16.1 (1)**	12.2 (4)	**15.3 (1)**	**17.5 (3)**	12.7 (4)	**17.4 (1)**	**28.6 (1)**	**24.5 (1)**	**34.5 (1)**	**34.7 (1)**	**27.3 (1)**	**37.9 (1)**
hypertension&cholesterol&osteoarthrosis	**16.0 (3)**	**21.0 (2)**	11.0 (4)	**15.5 (2)**	18.9 (6)	**24.7 (3)**	10.4 (6)	11.9 (6)	**13.5 (2)**	**18.9 (2)**	7.4 (26)	**16.0 (2)**	18.0 (7)	19.0 (4)	20.6 (6)	**28.9 (2)**	17.1 (4)	**27.1 (2)**
cholesterol&osteoarthrosis&back pain	**17.6 (2)**	**20.3 (3)**	**11.7 (2)**	13.0 (6)	**21.1 (3)**	**25.2 (2)**	**13.6 (2)**	12.2 (5)	12.1 (4)	14.4 (6)	6.3 (32)	12.3 (8)	22.0 (4)	**22.7 (2)**	**22.3 (3)**	**28.4 (3)**	15.9 (7)	**24.5 (3)**
hypertension&cholesterol&back pain	12.2(10)	18.1 (4)	8.1 (8)	15.2 (4)	14.7(12)	20.1 (4)	9.5 (7)	**16.1 (3)**	8.5 (11)	14.7 (4)	3.6 (96)	14.2 (5)	14.8 (11)	16.8 (7)	16.2 (12)	22.6 (4)	10.8 (17)	21.3 (5)
hypertension&cholesterol&diabetes	6.6 (43)	14.3 (5)	6.2 (21)	**17.9 (1)**	6.8 (64)	11.8(19)	6.0 (18)	**17.2 (1)**	6.9 (18)	20.5 (1)	5.0 (51)	**14.9 (3)**	6.7 (61)	7.6 (39)	7.3 (64)	13.4 (18)	5.8 (72)	15.3 (18)
osteoarthrosis&back pain&varicose veins	15.9 (4)	13.9 (6)	5.8 (26)	5.3 (38)	**21.8 (2)**	19.8 (5)	7.1 (15)	4.8 (35)	5.2 (47)	5.6 (46)	3.9 (85)	5.7 (45)	**23.5 (2)**	17.6 (5)	**22.4 (2)**	20.3 (5)	16.2 (6)	22.3 (4)
obesity&osteoarthrosis&back pain	13.8 (7)	13.2 (7)	8.9 (7)	7.5 (18)	16.7 (8)	17.0 (7)	**12.6 (3)**	6.9 (17)	6.5 (23)	9.1 (17)	5.0 (53)	6.1 (44)	18.6 (6)	16.9 (6)	17.5 (8)	18.1 (8)	9.9 (19)	15.7 (14)
osteoarthrosis&back pain&mental health	15.2 (5)	13.0 (8)	6.3 (20)	4.4 (55)	20.3 (5)	18.9 (6)	7.6 (11)	3.4 (71)	5.3 (46)	5.1 (56)	5.3 (46)	5.0 (59)	**22.2 (3)**	**19.1 (3)**	19.8 (7)	18.0 (9)	**17.3 (3)**	19.7 (7)
hypertension&obesity&osteoarthrosis	13.7 (8)	13.0 (9)	9.9 (5)	8.8 (11)	15.9 (9)	15.9(10)	10.6 (5)	8.0 (15)	10.7 (6)	11.2(10)	6.3 (33)	6.3 (42)	16.3 (9)	14.7 (9)	17.4 (10)	17.9 (10)	11.5 (15)	14.9 (20)
hypertension&diabetes&osteoarthrosis	9.3 (17)	12.2(10)	6.7 (15)	9.8 (9)	10.7(22)	13.8(12)	5.3 (30)	6.7 (18)	7.3 (13)	12.1 (9)	8.9 (16)	11.5 (11)	9.1 (36)	8.4 (32)	12.9 (17)	15.7 (11)	9.3 (23)	18.7 (10)
hypertension&cholesterol&obesity	7.4 (30)	12.1(11)	7.1 (12)	13.2 (5)	7.6 (52)	11.3(23)	9.4 (8)	**16.4 (2)**	6.9 (17)	14.4 (5)	1.7(173)	5.2 (52)	7.9 (52)	10.9 (17)	8.5 (49)	12.1 (25)	4.9 (95)	11.0 (32)
osteoporosis&back pain&prostate (men)	-	-	**11.7 (3)**	7.3 (20)	-	-	11.0 (4)	4.7 (37)	10.9 (5)	7.5 (24)	**14.8 (1)**	11.7 (9)	-	-	-	-	-	-
							< prim		prim/lo	sec	hi sec	voc/uni	< prim		prim/lo	sec	hi sec	voc/uni
							2006	2017	2006	2017	2006	2017	2006	2017	2006	2017	2006	2017
hypertension&osteoarthrosis&back pain							**19.3 (1)**	**18.2 (3)**	**13.7 (1)**	**16.3 (2)**	**13.1 (1)**	10.7 (6)	**33.7 (1)**	**39.5 (1)**	**30.9 (1)**	**29.8 (1)**	**18.4 (2)**	**20.1 (1)**
hypertension&cholesterol&osteoarthrosis							14.5 (4)	**19.0 (1)**	9.3 (5)	15.4 (4)	10.5 (4)	12.1 (4)	21.3 (5)	**31.6 (2)**	18.4 (7)	**22.1 (3)**	11.3 (8)	**16.9 (3)**
cholesterol&osteoarthrosis&back pain							13.3 (5)	15.2 (5)	**11.1 (2)**	13.3 (5)	**11.0 (2)**	10.3 (8)	**23.7 (3)**	**30.1 (3)**	20.1 (5)	**23.2 (2)**	**15.2 (3)**	**20.0 (2)**
hypertension&cholesterol&back pain							11.6 (9)	17.9 (4)	5.8 (21)	**15.9 (3)**	8.9 (5)	10.7 (5)	17.3 (11)	24.4 (4)	13.7 (14)	18.5 (5)	8.8 (17)	14.9 (4)
hypertension&cholesterol&diabetes							6.9 (31)	**18.4 (2)**	6.6 (12)	**18.5 (1)**	3.9 (62)	**16.4 (1)**	9.9 (41)	16.9 (16)	5.5 (78)	9.9 (21)	1.0 (183)	5.8 (52)
osteoarthrosis&back pain&varicose veins							5.9 (48)	6.9 (48)	5.6 (24)	5.3 (34)	6.1 (18)	3.6 (57)	**23.9 (2)**	24.0 (5)	**21.5 (2)**	18.9 (4)	14.4 (4)	12.8 (5)
obesity&osteoarthrosis&back pain							12.1 (8)	10.6(15)	7.6 (7)	7.7 (17)	7.8 (6)	4.3 (41)	20.4 (6)	22.0 (8)	15.7 (8)	15.6 (8)	6.1 (33)	9.6 (16)
osteoarthrosis&back pain&mental health							6.7 (38)	6.3 (57)	6.5 (13)	4.4 (51)	5.1 (32)	2.6 (97)	21.7 (4)	23.8 (6)	20.5 (4)	17.5 (6)	14.1 (5)	11.7 (7)
hypertension&obesity&osteoarthrosis							13.1 (6)	11.1(12)	10.0 (4)	9.1 (14)	4.7 (41)	6.0 (20)	19.0 (9)	20.9 (10)	15.3 (9)	14.1 (9)	6.3 (30)	9.4 (17)
hypertension&diabetes&osteoarthrosis							8.7 (17)	13.3 (7)	7.2 (8)	9.2 (13)	2.7 (94)	7.4 (10)	13.8 (18)	21.2 (9)	9.2 (30)	11.2 (17)	5.8 (40)	4.6 (77)
hypertension&cholesterol&obesity							8.5 (20)	13.2 (8)	6.0 (16)	13.2 (6)	7.7 (8)	**13.0 (3)**	10.5 (35)	14.1 (28)	6.2 (65)	9.7 (23)	3.2 (103)	10.0 (13)
osteoporosis&back pain&prostate (men)							14.6 (3)	10.9(14)	**10.4 (3)**	6.7 (23)	**10.8 (3)**	4.9 (33)	-	-	-	-	-	-

Supplementary Table S3. Ten-highest Observed/Expected (O/E) ratios of 3-way disease combinations of chronic conditions and their prevalence and rank among 60-89 year-old people living with multimorbidity in Spain. 2006 and 2017.

	2006	
Combination	O/E (Rank)	Prevalence (Rank)
haemorrhoids&gastric ulcer&constipation	4.4 (1)	0.9 (688)
urinary incontinence&prostate problems&skin problems	4.2 (2)	1.0 (648)
migraine&gastric ulcer&constipation	4.1 (3)	1.0 (646)
COPD/asthma&alergies&skin problems	3.8 (4)	0.9 (696)
mental health&migraine&gastric ulcer	3.5 (5)	1.4 (466)
haemorrhoids&migraine&constipation	3.5 (6)	1.4 (473)
alergies&gastric ulcer&constipation	3.5 (7)	0.6 (853)
urinary incontinence&haemorrhoids&constipation	3.5 (8)	1.3 (495)
haemorrhoids&osteoporosis&constipation	3.4 (9)	1.7 (386)
urinary incontinence&prostate problems&gastric ulcer	3.4 (10)	0.6 (835)

	2017	
Combination	O/E (Rank)	Prevalence (Rank)
haemorrhoids&migraine&constipation	5.8 (1)	0.9 (690)
haemorrhoids&osteoporosis&constipation	5.5 (2)	1.2 (561)
haemorrhoids&mental health&constipation	5.4 (3)	1.9 (367)
haemorrhoids&gastric ulcer&constipation	4.9 (4)	0.4 (959)
mental health&gastric ulcer&constipation	4.6 (5)	0.8 (755)
urinary incontinence&haemorrhoids&constipation	4.5 (6)	1.2 (579)
migraine&gastric ulcer&constipation	4.4 (7)	0.4 (1005)
haemorrhoids&skin problems&constipation	4.2 (8)	0.7 (834)
gastric ulcer&skin problems&constipation	4.2 (9)	0.4 (1011)
urinary incontinence&osteoporosis&constipation	4.0 (10)	1.3 (532)

Supplementary Table S4. The highest factor loadings from the PCA of each chronic condition (>0.3), communalities, eigenvalues and % cumulative variance of components for the multimorbidity sample (ages 60-89). 2016 and 2017. Total population aged 60-89 and by sex.

**a. Total population**	2006									2017						
	Component								Commu-nality	Component						Commu-nality
**Chronic condition**	1	2	3	4	5	6	7	8		1	2	3	4	5	6	
Hypertension						0.510			0.380			0.603				0.391
Cholesterol								0.570	0.419			0.584				0.517
Heart disease								0.672	0.564					0.385		0.279
Diabetes						0.444			0.348			0.613				0.408
COPD/asthma				0.714					0.597				0.534			0.442
Urinary Incontinence			0.779						0.679					0.611		0.489
Haemorrhoids					0.784				0.630		0.643					0.436
Thyriod dysfunction							0.651		0.489							0.268
Obesity						0.709			0.619						0.751	0.599
Osteoporosis	0.528								0.395	0.528						0.381
Osteoarthrosis	0.721								0.546	0.752						0.583
Chronic back pain	0.708								0.536	0.659						0.473
Mental health problems		0.622							0.454		0.516					0.322
Migraine		0.567							0.358		0.339					0.191
Prostate			0.671						0.601					0.572		0.508
Varicose veins							0.576		0.393						0.331	0.260
Alergies				0.672					0.517				0.692			0.543
Gastric and duodenal ulcer		0.604							0.520		0.354					0.214
Chronic skin problems				0.304					0.328				0.582			0.373
Constipation					0.674				0.496		0.606					0.400
Eigenvalue	1.933	1.420	1.191	1.166	1.081	1.048	1.021	1.009		2.091	1.378	1.253	1.165	1.126	1.067	
% cumulative variance	9.7	16.8	22.7	28.5	34.0	39.2	44.3	49.3		10.5	17.3	23.6	29.4	35.1	40.4	

Supplementary Table S5. Agglomeration schedule of the hierarchical clustering using centroid linkage and Yule's Q similarity

2006

	Cluster combined				appears		
Stage	Cluster 1	Cluster 2	Coefficients	Change	Cluster 1	Cluster 2	Next stage
1	7	20	0.577		0	0	13
2	11	12	0.516	0.061	0	0	6
3	6	15	0.472	0.044	0	0	19
4	5	17	0.427	0.045	0	0	8
5	13	14	0.360	0.067	0	0	10
6	10	11	0.350	0.011	0	2	16
7	1	4	0.235	0.115	0	0	11
8	5	19	0.185	0.050	4	0	17
9	8	16	0.183	0.002	0	0	15
10	13	18	0.142	0.041	5	0	13
11	1	2	0.073	0.069	7	0	12
12	1	3	0.027	0.046	11	0	14
13	7	13	0.013	0.014	1	10	15
14	1	9	-0.023	0.035	12	0	18
15	7	8	-0.034	0.012	13	9	16
16	7	10	-0.060	0.025	15	6	17
17	5	7	-0.101	0.041	8	16	18
18	1	5	-0.193	0.092	14	17	19
19	1	6	-0.184	-0.008	18	3	0

2017

	Cluster combined				appears		
Stage	Cluster 1	Cluster 2	Coefficients	Change	Cluster 1	Cluster 2	Next stage
1	7	20	0.631		0	0	10
2	11	12	0.537	0.094	0	0	6
3	6	15	0.436	0.101	0	0	13
4	5	17	0.384	0.052	0	0	9
5	13	14	0.362	0.023	0	0	11
6	10	11	0.348	0.014	0	2	16
7	1	4	0.316	0.032	0	0	8
8	1	2	0.210	0.106	7	0	15
9	5	19	0.208	0.002	4	0	17
10	7	18	0.202	0.006	1	0	12
11	13	16	0.130	0.072	5	0	12
12	7	13	0.061	0.070	10	11	14
13	3	6	0.059	0.001	0	3	18
14	7	8	0.014	0.046	12	0	16
15	1	9	-0.024	0.037	8	0	18
16	7	10	-0.056	0.032	14	6	17
17	5	7	-0.095	0.039	9	16	19
18	1	3	-0.137	0.042	15	13	19
19	1	5	-0.214	0.076	18	17	0

Note: The coefficients that are shown in the agglomeration schedule are related to the distances at which clusters are combined. Although there is no clear moment when there is a sudden increase in the coefficient in either year, differences are somewhat higher from stage 14 onwards in both years. Moreover, most clusters that are obtained up until this point can be considered clinically meaningful (see Supplementary Figure 3).

Supplementary Figure S1. Average number of conditions among persons living without and with multimorbidity by sex, age and educational level. 2006 and 2017.

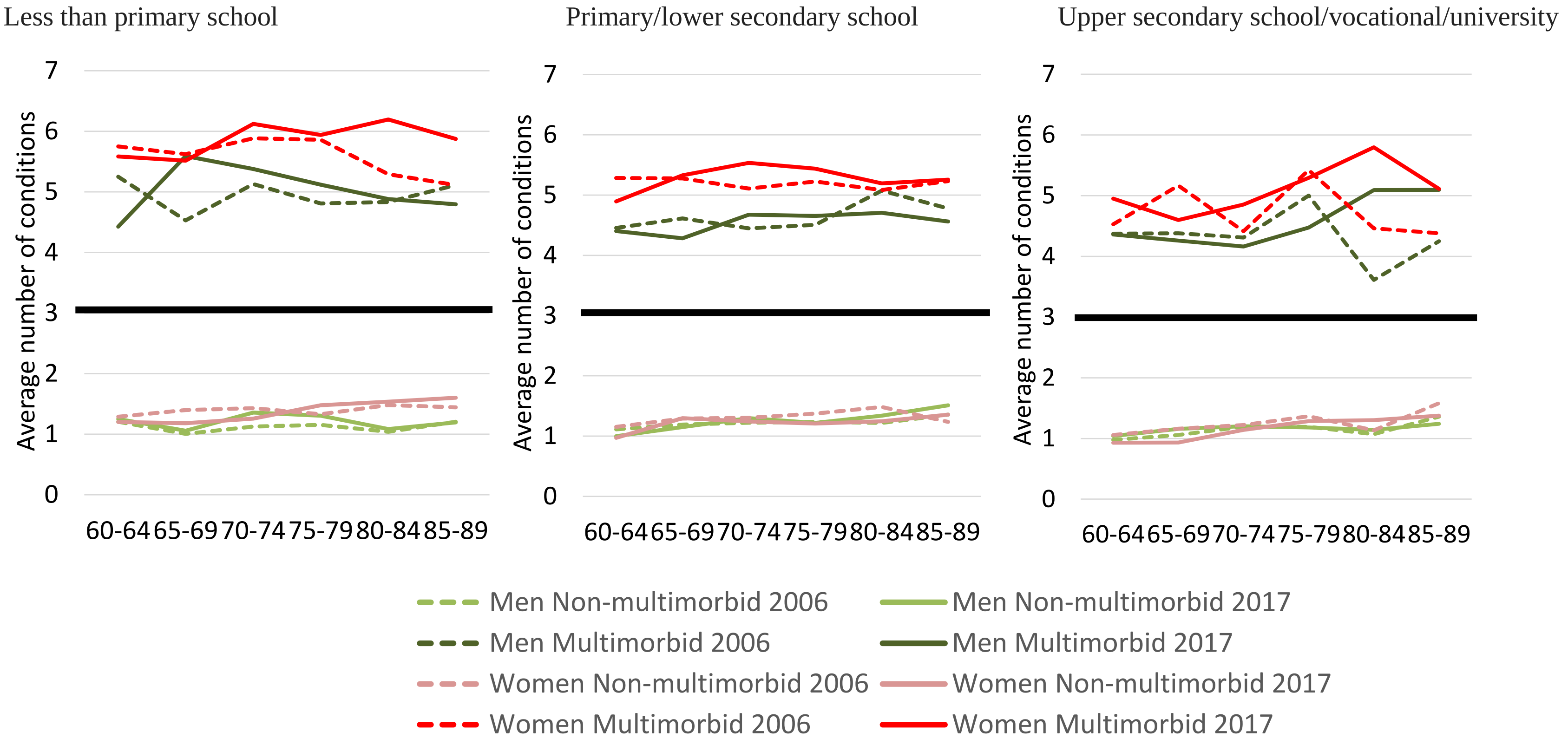

Supplementary Figure S2. Scree plots of PCA analysis. 2006 and 2007.

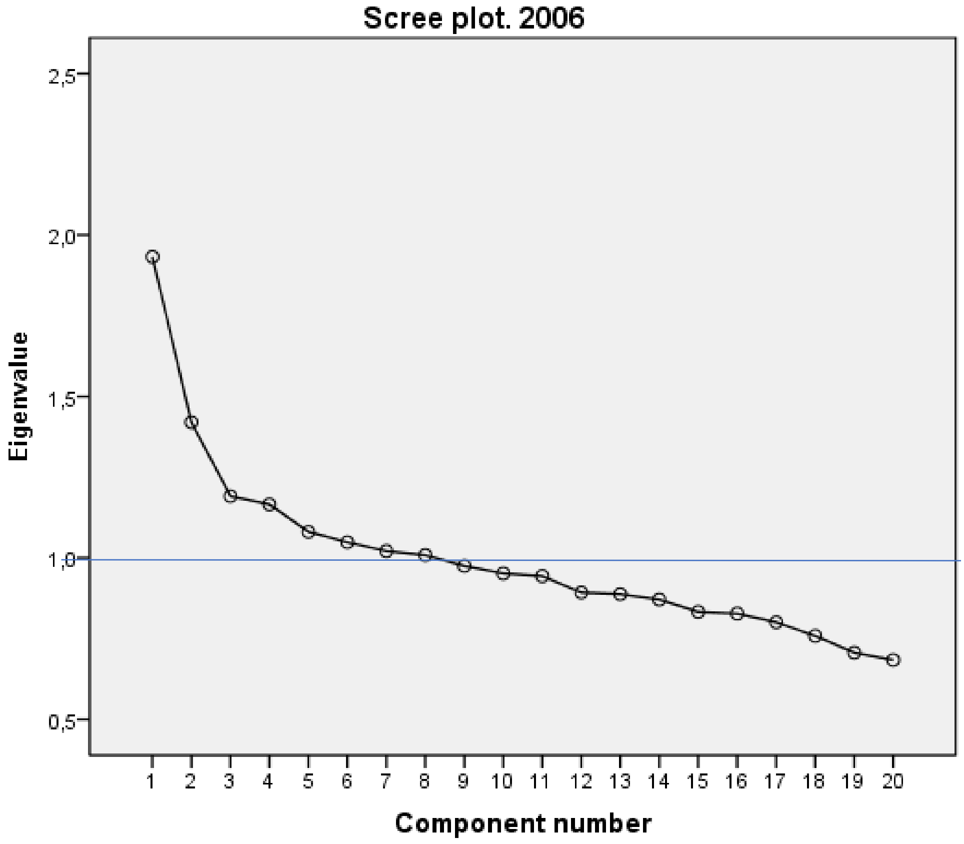

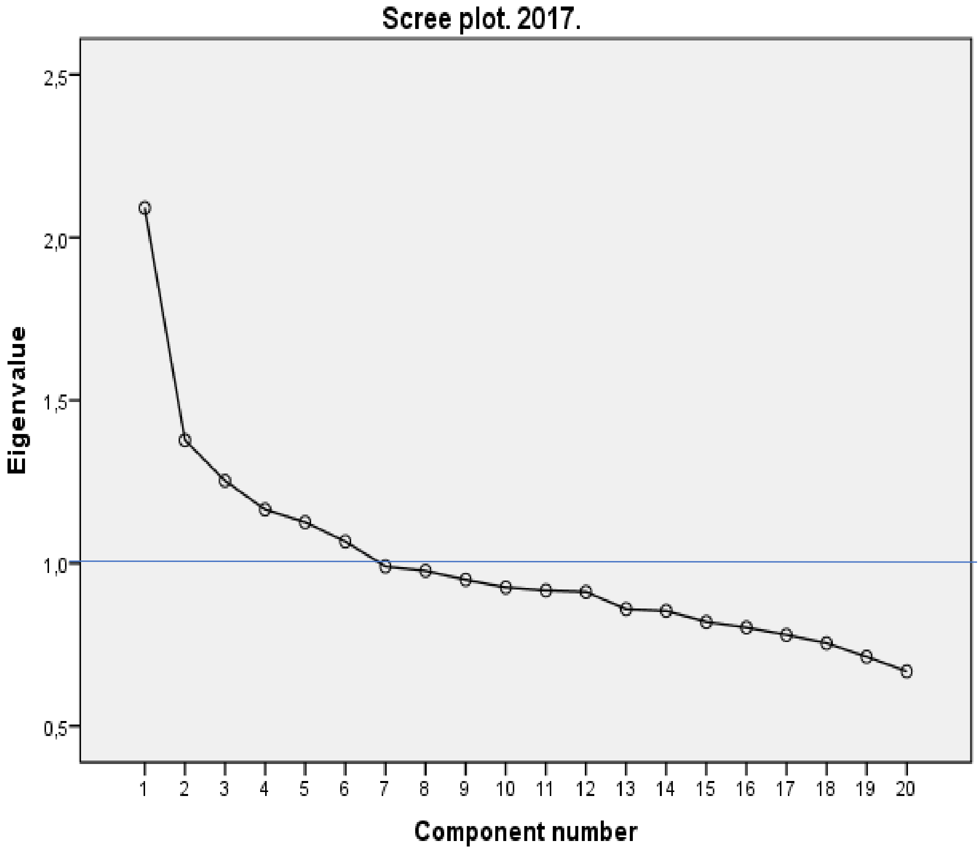

Supplementary Figure S3. Dendrogram of Cluster Analysis using centroid linkage and Yule's Q similarity

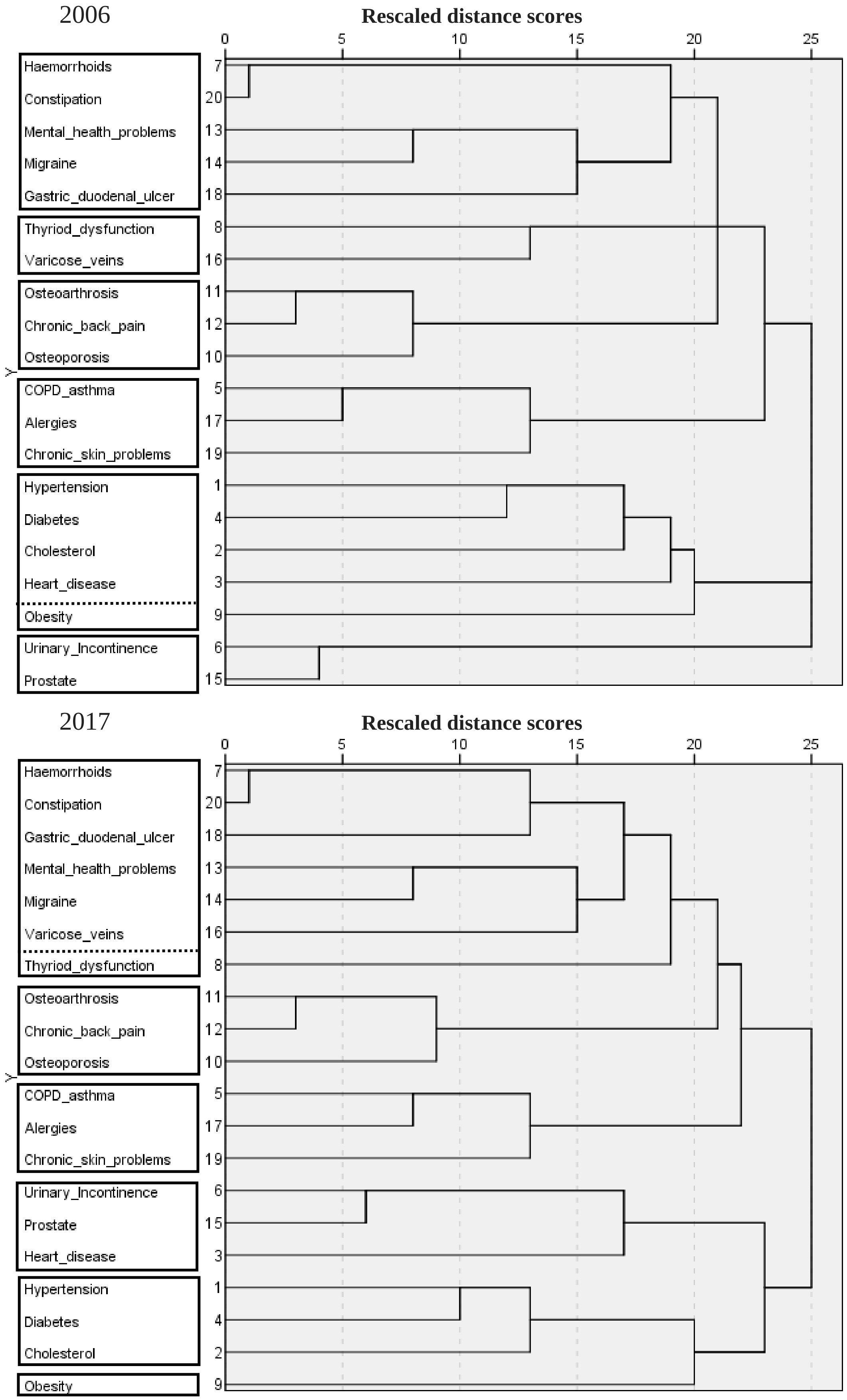

Note: The agglomeration schedule of the hierarchical clustering can be found in Supplementary Table 4.
